# Psychological Impact of Presymptomatic X-Linked ALD Diagnosis and Surveillance: A Small Qualitative Study of Patient and Parent Experiences

**DOI:** 10.3390/ijns10040073

**Published:** 2024-10-24

**Authors:** Cecilie S. Videbæk, Sabine W. Grønborg, Allan M. Lund, Mette L. Olesen

**Affiliations:** 1 Department of Inherited Metabolic Diseases, Copenhagen University Hospital Rigshopitalet, 2100 Copenhagen, Denmark; sabine.groenborg@regionh.dk (S.W.G.); allan.lund@regionh.dk (A.M.L.); 2 Department of Gynecology, Copenhagen University Hospital Rigshopitalet, 2100 Copenhagen, Denmark

**Keywords:** adrenoleukodystrophy, XALD, disease-related loneliness, presymptomatic diagnosis

## Abstract

X-linked adrenoleukodystrophy (ALD) is a rare metabolic disorder. Symptoms range from cerebral demyelination (cALD) to adrenal insufficiency and slowly progressive myeloneuropathy. cALD is fatal if not treated with hematopoietic cell transplantation in the early stages of the disease course. This can be achieved through cascade testing or newborn screening (NBS). Due to the lack of predictive measures of disease trajectory, patients are monitored with frequent MRI scans and hormone testing to ensure timely intervention. With this study, we wanted to explore how the diagnosis of ALD, before the development of cALD, and the follow-up program affected patients and their parents. Using semi-structured interviews, we interviewed seven parents of children with ALD aged 3–11 and four patients with ALD aged 18–25. Because NBS for ALD has not been implemented in Denmark, the patients were identified through either cascade testing or after having presented with adrenal insufficiency. We generated five themes: (I) ALD patients maintained mental resilience despite diagnosis and surveillance; (II) patients’ concerns matured with age and centered around situations that confronted them with their patient status; (III) parents of children with ALD had both short-term and long-term worries for their children’s health; (IV) parents took on a huge psychological burden; and (V) due to its rarity, the diagnosis of ALD evoked a sense of isolation and disease-related loneliness. Overall, we found a large discrepancy in the experiences reported by parents and patients. Despite the small sample size, we identified patterns that suggest that while the early diagnosis took a significant psychological toll on the parents, patients lived relatively carefree lives despite their ALD diagnosis.

## 1. Introduction

X-linked adrenoleukodystrophy (ALD) is caused by pathogenic variants in the *ABCD1* gene, leading to impaired peroxisomal ß-oxidation of saturated straight-chain very long-chain fatty acids (VLCFAs), resulting in VLCFA accumulation in the plasma and tissues [1]. The incidence has been reported as between 0.8 and 6 per 100,000 in epidemiological studies [2]. ALD is an X-linked disorder and presents with more severe symptoms in male patients. Symptoms range from severe, fatal cerebral demyelination (cALD) in childhood (40%) or adulthood (10%) [3] to adrenal insufficiency (80% in childhood) [3]. Furthermore, almost all male patients [3] and approximately 80% of female patients [4,5] will develop slowly progressive myeloneuropathy (AMN). More than 1040 pathogenic gene variants have been described [6], but no correlation between genotype and phenotype has been identified [7].

Treatment of ALD depends on the phenotype. Patients with adrenal insufficiency are treated with hormone replacement therapy. Progression of cALD can be halted by hematopoietic cell transplantation (HCT) [8], but for the treatment to be successful, HCT must be performed in the very early disease stages [9]. Dietary restrictions and Lorenzo’s oil have been proposed as preventative options for XALD; however, their effects have not been confirmed [10].

To ensure timely treatment of adrenal insufficiency and cALD, patients who have been diagnosed presymptomatically are kept under surveillance throughout childhood and adulthood. This includes screening for Addison’s disease with hormone measurements or ACTH-stimulation tests every 3–12 months (depending on age) and cerebral magnetic resonance imaging (MRI) every six months during childhood and every 12 months in adulthood [9].

Given the potential benefits of early diagnosis and treatment of ALD, the state of New York became the first to implement newborn screening (NBS) for ALD, following the passage of Aidens Law in 2013. Since then, 44 American states, Taiwan [11], and the Netherlands [12] have included ALD in their NBS program. Japan [13], Italy [14], and China [15] currently have ongoing pilot studies.

While early detection and surveillance enable potentially lifesaving treatment of ALD, they also necessitate extensive follow-up. Due to the lack of prognostic biomarkers, predicting the disease course for individual patients with ALD is impossible, leading to uniform extensive follow-up for all pre-symptomatic males diagnosed with the condition. Several studies have established that there is a positive attitude toward presymptomatic testing and diagnosis of ALD in the ALD patient community [16,17,18,19] despite the surveillance program. We know from other groups diagnosed with genetic risk factors that while this knowledge has the potential to be lifesaving, follow-up programs can also impose a significant burden [20,21]. Furthermore, it is known that follow-up of children who survive childhood brain cancer puts a significant toll on families [22,23].

The possibility of implementing NBS for ALD in Denmark is being explored. We conducted this study to investigate the impact of the surveillance program on ALD patients and their families, gain insight into the psychological aspects, and identify possible ways to support these patients and families. We hope that the results can help to optimize the follow-up of ALD patients diagnosed before the onset of cALD through cascade testing or NBS.

## 2. Aim

This study aimed to explore the experiences of being diagnosed with ALD, attending the regular ALD surveillance program before the onset of cALD, and living with ALD for both young patients and their parents.

## 3. Materials and Methods

### 3.1. Study Design

We conducted a qualitative study using semi-structured interviews. The study was conducted and reported according to the consolidated criteria for reporting qualitative research (COREQ) [24].

### 3.2. Setting

Interviews were performed between May 2023 and February 2024 at an undisturbed location at the Center for Inherited Metabolic Diseases, Copenhagen University Hospital, on the same day as a planned routine visit. Two interviews were conducted virtually.

### 3.3. Participants

Due to the rarity of ALD, we used convenience sampling [25]. In a given patient, all inclusion criteria should be fulfilled, and they were: (I) patients with an ALD diagnosis verified by genetic testing and raised plasma levels of VLCFA; (II) male patients; (III) patients aged 0–30 years; (IV) patients spoke Danish or English; and (V) patients attending ALD surveillance at Copenhagen University Hospital according to current guidelines. Patients were not interviewed if they were under the age of eight.

We aimed to interview the parents of eligible patients aged 0–18. The only other inclusion criteria was for parents to speak Danish or English. Treating physicians screened for eligibility. We chose to include the parent of one patient who had recently undergone HCT.

### 3.4. Semi-Structured Interviews

We schematized aspects of disease knowledge, disease burden, and the burden of the follow-up program in a semi-structured interview guide. A translated version of the interview guide is provided in Supplementary Tables S1 and S2. We pilot-tested our interview guide; however, due to our small sample size and the absence of substantial changes to the guide, we chose to include the pilot study in the data analysis. Following the pilot interview, we changed the order of questions one and two to ensure a more natural flow. Additionally, based on insights from the pilot interview, we incorporated one new question into the interview guide (indicated by italics in Supplementary Tables S1 and S2). This addition aimed to either confirm or refute specific aspects that surfaced during the pilot. Furthermore, one question about loneliness (marked with italics in Supplementary Tables S1 and S2) was added after the first interview of parents. Interviews lasted between 27 and 51 min. All interviews were conducted by CV, who had no relationship with the participants before the interview. Only the participants and the interviewer were present at the interview. No interviews were repeated. All interviews were audio-recorded and transcribed verbatim by CV. Transcribed interviews were not returned to the participants. Field notes were made both during and after the interviews to document the context, emotions, and nuances in the interviews but also to keep track of practical matters such as changes made to the interview guide, data saturation (where no significant new information emerged), etc. Despite the relatively small cohort, we saw a pattern that could suggest data saturation after the third patient interview and fourth parent interview, where answers to our interview questions became repetitive of the previous.

### 3.5. Ethics

The study was approved by the Scientific Ethics Committee in the Central Region of Denmark (H-23007728). The study was conducted according to the Declaration of Helsinki.

All participants received preliminary information about the study from their treating physician, who was a doctor who had followed the family for years, and with whom the families were familiar. The participants were informed about the study by telephone and sent written information by post. For patients under the age of 18, the parents were approached. All participants provided informed written consent.

### 3.6. Data Analysis

For the data analysis, we used inductive, thematic analysis, as described by Braun and Clarke [26]. This included the following five steps: (I) getting familiar with the data; (II) generating the initial codes; (III) gathering codes into potential themes; (VI) reviewing the themes; (V) defining and naming themes; and (VI) producing a report of the analysis. First, all data were read by CV and MLO and initial codes were written in the margins. The data were then transferred to the qualitative data analysis software NVivo (version 14.23.0), where they were coded into the preliminary codes. Through continuous dialog, mind maps, and reflection, the data were organized manually into themes by CV and MLO. After the initial themes had been identified, all data were recoded using NVivo to reflect the identified themes by CV and MLO. The final phase included a discussion among all authors to agree on the five key themes. Finally, we returned to our transcribed data, reassured that the identified themes reflected the overall meaning of the dataset. All quotes were translated into English and adjusted so each parental quote pertains to a single child. The latter was performed to protect the identity of parents with multiple children affected by ALD. Examples of the process are shown in the code tree in Table 1.

## 4. Results

A total of four patients and seven parents were interviewed. The demographic details of participants can be found in Table 2. Participants are described in groups to preserve their anonymity.

Five overall themes were generated. Two themes covering patient experiences:

“ALD patients maintained mental resilience despite diagnosis and surveillance” and “Patients’ concerns matured with age and centered around situations that confronted them with their patient status”.

While two themes covered the parent’s experience:

“Parents of children with ALD had both short-term and long-term worries for their children’s health” and “Having a child with ALD was a big psychological burden for parents”.

One overall theme covering a common experience was also generated: “Due to its rarity, the diagnosis of ALD evoked a sense of isolation and disease-related loneliness”. See Table 3 for an overview of themes and subthemes.

### 4.1. ALD Patients Maintained Mental Resilience Despite Diagnosis and Surveillance

In this theme, patients described that the diagnosis of ALD did not affect their day-to-day life. The theme contains the following two subthemes.

*Patients aspired to lead as normal lives as possible*

We found that patients sought normality in their lives. They actively avoided letting their ALD diagnosis compromise them and generally maintained a positive attitude toward their future with ALD. Patients also described a desire not to be perceived as compromised by their condition: “*At one time, many started treating me differently, and that actually made me angry. Why should I be treated any different than others? I don’t want that. I don’t want to be that freak in the corner*”. (Patient 3) and “*I mean talk briefly with my friends about it, but I don’t want to be seen as the victim in the group, so I try to keep it as minimal as possible*”. (Patient 4). While most were not opposed to discussing their diagnosis if asked, a patient explained avoiding disclosing his diagnosis to others.

Another example of patients striving to lead as normal lives as possible was illustrated by their choices of careers and lifestyles. While having the knowledge that they might someday develop neurological symptoms, this did not affect their future planning, as described below: “And then I probably have a job, that I shouldn’t have. I am a * [a physically demanding job], which is beyond all limits. I think, I mean, I have plans for the future with it, later I might have to find an angle to it where I don’t have to be in the field so much”. (Patient 3).

Patients in general did not seem very occupied with their diagnosis. They were aware of the basics concerning ALD phenotypes but were not concerned about details about the pathophysiology, ongoing clinical trials, or treatment options. They generally chose to remain hopeful and positive about their diagnosis but were not oblivious to the potential consequences: “*Maybe it makes you think, that if you want to do something in your life, then perhaps you should do it a little earlier. Like if you want to run a marathon or travel around the world, then perhaps you shouldn’t wait until you are 50*”. (Patient 2).

*Patients had great trust in healthcare professionals and it gave them an opportunity to leave their own worries behind*

It was evident from the data that the annual consultations in the surveillance program did not cause distress for ALD patients in general or in the time leading up to the consultation, in both childhood and adulthood. Thinking back on his childhood, a patient reminisced: “*As a child, I just thought it was great coming here and getting tests done, and getting compliments, and being told: “Okay! You are actually really strong”. I think that was actually what I focused on the most*”. (Patient 4). This sentiment was also backed up by the parents, parent 4 said: “He’s just like “Wuhuu are we seeing * [the patient doctor] today? […] There are never any complaints, honestly, I think he just enjoys getting the day off from school”. None of the patients described fear in relation to MRI results.

They exhibited a strong trust in the healthcare professionals who had monitored them and in the surveillance program itself. This trust allowed them to focus on other aspects of their daily lives, enabling their feeling of normalcy: “Well when I came to * [Doctor’s name] and they performed all the tests, I felt, like, really safe. Because, I kinda felt, that there were professionals who had taken care of it. I mean, I knew I had a disease, so I felt that it was a good place [the Department of Inherited Metabolic Diseases] and that it was important that I was there and that it was good and safe to be there”. (Patient 1).

### 4.2. Patients’ Concerns Matured with Age and Centered Around Situations That Confronted Them with Their Patient Status

Even though patients aspired to live as normal lives as possible, they were sometimes in situations where they could not avoid their disease, which led to some concerns. These concerns mainly centered around concrete and often unavoidable aspects of living with the ALD diagnosis. The theme included three subthemes:

*In childhood, patients primarily expressed concerns about concrete situations that were unavoidable due to their disease.*

It became apparent that the patients’ concerns were dynamic and changed with age. During childhood, the primary worries for ALD patients revolved around tangible issues, such as painful experiences like blood draws, IV insertions, and anesthesia before MRI scans. In the following, a patient reflected on his childhood: “When I was younger it was a bit overwhelming… I mean being put under anesthesia, that wasn’t for me when I was younger… But I mean, of course, I wasn’t very old and I didn’t understand why it happened and why I had to be put under anesthesia… But I knew I was in good hands”. (Patient 1) and “While concerns about painful procedures were prominent at the moment, they typically had little impact on the children in the time around the procedure. A parent described “But every time we leave he is happy. It’s like he also thinks “okay it’s over” and then it’s out of his head. I mean, I don’t think we have tried not to leave with a happy child yet. Because it’s just like … well… then it disappears”. (Parent 2). Parent 7 proposed using visuals to assist in preparing the children for upcoming procedures during follow-up appointments. He argued that, in his experience, it was easier for his son to prepare himself for painful procedures or to lie very still if he had seen visual representations of what was going to happen.

Dietary restrictions, particularly evident during social events like birthdays, also worried the children and made them feel different from their peers. All patients on dietary restrictions described a sense of isolation and worry about situations where they were confronted with foods they were not allowed to eat. “It is mentally really hard. With me, when I was younger, my parents were really strict about what I could eat… If there was cake at school, I was taught not to eat it, and the teachers were also told *[that I couldn’t eat cake]. If there was a birthday and I wasn’t allowed to eat anything… that was really, really difficult*”. (Patient 2), a patient reminisced.

*The transition from child to adult was difficult.*

The teenage years were the most challenging for the patients, as they had to assume greater responsibility for managing their disease and taking charge of aspects such as dosing of medicine and complying with dietary restrictions which were previously overseen by their parents. A patient described “*I have had periods where it [the diagnosis] took up more mental space than it should, where it was hard to think about. I think it was around the age of 15–16 when I started partying and I had to take responsibility for it myself, that I started thinking a lot about it*”. (Patient 4). Patients also started spending more time with friends and had to decide how much to involve them in their disease. For teenagers on dietary restrictions, parental control over their food choices became more difficult to enforce, and they were not always diligent about following the prescribed diet. A patient said “*After I moved out, I still followed the diet, but maybe not as strictly as when I was a boy. That can be a bit… but now I have started following it more again because there is more stability in my daily life. But yeah, it was hard mentally*”. (Patient 2).

*Adult patients’ predominant concern arose when faced with the prospect of passing ALD to their children*

As adults, all patients expressed concern about the disease when discussing the aspect of having children. They were all concerned about the potential of passing the disease on to their own children. “*The only thing that affects me is the thought about having children [with ALD] someday, and that we just have to accept. But that’s the only thing, and we’re not sitting around thinking “ohh why do we have to do that” when it’s not necessary right now—we’ll take that step when we get there*”. (Patient 3). While all patients were aware that actions would be necessary regarding family planning, they had not actively sought information on the specifics. Only one patient had considered the implication that he would not be able to father a daughter who would not be a carrier of ALD.

### 4.3. Having a Child with ALD Was a Big Psychological Burden for Parents

Having a child with ALD was a significant psychological burden for the parents who had major concerns for their children’s health. A parent opened up about this: “*It’s difficult for me with his disease… And it has also caused me some periods of stress […] It takes up a lot of my thoughts*”. (Parent 7). We found that although parents had many concerns themselves, they shielded their children from as much as possible regarding ALD. This theme consists of three subthemes.

*Parent’s worries were worst in the time around hospital visits and MRI*

Parents experienced that their worries were notably worse in the time around hospital visits. A parent described: “It hits as soon as we start getting reminders in “Min Sundhed” *[The platform used for communicating with the healthcare system in Denmark]*, that’s when you get reminded… I find that I’m actually pretty good at not thinking about it in between *[visits]*… that’s about 4 months and then you’re reminded again”. (Parent 3). Especially, the MRI results worried the parents, they described that they had less energy, lost track of things, and felt “jumpy” in the time leading up to the MRI scans. Several parents described planning these follow-up visits at specific times during the year so that it did not interfere with holidays or special occasions. The worst part seemed to be the period between undergoing the MRI and receiving the results. During this period, parents described being glued to their phones and having difficulty concentrating on work or other tasks. Once a positive result had been given, they felt a huge relief and could let go of their worries for a while: “It’s even worse when you’re waiting for results, and then when you get the results and they’re positive, you completely relax again. But then you also know that there will be another period where you’ll have to wait for new results again”. (Parent 4). Parents also described receiving the ALD diagnosis as a massive stressor in itself: “It was an unbearable shock when we were told back when he was * [patients age at diagnosis]. We were really, I would say almost traumatized from it. Also because you’re in a phase where you are not really very well prepared to deal with this…” (Parent 1). Because the disease and continuous follow-up were so psychologically straining for the parents, parents 5 and 7 suggested that therapist sessions for the parents should be part of the standard surveillance program at the first follow-up sessions.

*Parents coped by being well-informed about ALD*

All parents had extensive knowledge about the ALD diagnosis. They all understood the genetic basis, pathophysiology, disease spectrum, and treatment options. They were well-informed about current ongoing trials, and future research questions as well as the consideration of NBS. Parents had a good understanding of the surveillance program and the reasoning behind each test that their children went through. Furthermore, they had a good understanding of their children’s results and were aware of specific concerns, such as a slight decrease in sensitivity shown in the corticotrophin stimulation test.

*Parents avoided telling their children the difficult parts and carried this burden alone*

Although the parents were very well-informed about ALD, they were very strategic with the information they chose to share with their children. Because of the considerable uncertainty surrounding it, they often opted to withhold key information from their children to shield them. This decision was typically rooted in the belief that there was no need to unnecessarily worry their children until the disease course became clearer. A parent said “We talk a lot about what is going to happen [talking about the procedures in the surveillance program], but not really why he has to or what comes from it… And we haven’t really talked about what the consequences are of getting a bad result from the scan. He doesn’t ask either. At some point, he probably will, and at that time we will have to make up our minds about how much we want to refer to his * [an affected relative’s] disease course. But otherwise, we’re taking it one step at a time and we try not to put words into his mouth or thoughts in his head that aren’t necessary”. (Parent 2). As patients grew older, parents struggled to decide when to start sharing more information with the affected child. Additionally, parents with older siblings faced the added challenge of deciding how much to disclose to them without risking that the information would be passed on to the affected sibling. A parent explained: “I mean, they ask a lot [the siblings] and we try to be open, but we haven’t told them about the serious stuff that can happen, just that we are watching him like * [an affected relative]”. (Parent 3). Parents tried to maintain optimism around all of their children and preserve their normal lives for as long as possible, to shield them from the psychological burden of the ALD diagnosis: “*It is also important to us that he’s allowed to be a completely normal child who doesn’t have to worry about anything. So we decided that when he asks about something, we’ll answer and try to give him the knowledge he needs. But otherwise, he doesn’t need to think about it—we’ll do that for him*”. (Parent 4).

### 4.4. Parents of Children with ALD Had Both Short-Term and Long-Term Worries for Their Children’s Health

Parents’ greatest and most predominant fear was the onset of cALD. The biggest fear concerning the onset of cerebral disease was the loss of their child. All parents described that this was what they struggled with the most. However, beyond the fatal consequences of onset of cALD, parents also had worries about their children undergoing HCT and consequently chemotherapy: “*Well if his brain is affected, then the treatment is a bone marrow transplantation. But we know, that it’s not 100% certain, that there is an effect, and sadly some children don’t make it…*” (Parent 5).

The parents described experiencing ambivalent feelings because they were thankful that the disease had been detected at a time when intervention against cALD was still possible, but the treatment itself also raised concerns. However, despite these ambivalent feelings, no parents expressed that they would have rather not known about their child’s ALD diagnosis before the onset of symptoms. Although it was not part of our interview guide, several parents spontaneously and independently described feeling lucky that the disease had been detected early enough for intervention to be possible. As one parent said: “*Well because it is really a terrible disease, but if we hadn’t known, then the boy wouldn’t have been checked and then he couldn’t be monitored and then it could have just gone terribly wrong*”. (Parent 4).

Although the fear of the onset of cALD was the most predominant, parents also had concerns about the future that their children could face with AMN. They struggled to find a balance, acknowledging the risk of their children developing severe myeloneuropathy with physical limitations, while also enjoying and encouraging their children’s active lifestyle and love for physical activities. Parent 2 reflected: “*We really focus a lot on what he can suffer from as a child and the bone marrow transplantation and all the things we’d have to go through if he had the changes detected in his brain… and then sometimes, I get hit by the thought that he can also really struggle as an adult… because we don’t know how this disease is going to develop and sometimes I think maybe I should steer him in the direction of a job that isn’t so physically demanding*”. (Parent 2). Parents also had more thoughts on preventative measures to ensure their children’s physical well-being for as long as possible: “*I have thought about that is important that he always stays active with some kind of workout or sport*”. (Parent 4).

### 4.5. Due to Its Rarity, the Diagnosis of ALD Evoked a Sense of Isolation and Disease-Related Loneliness

Both parents and patients struggled with the fact that ALD is such a rare and complicated disease. Parent 1 joked: “*Well, what can I tell you about the disease… I mean, I can’t even pronounce it!*” From the difficulty pronouncing its name to explaining its complex spectrum, families found it nearly impossible to inform friends and relatives about ALD, especially due to the lack of visible symptoms. The absence of symptoms makes it harder for others to understand the potential severity of the disease and understand the psychological burden on the families. Parents of children with adrenal insufficiency found it easier to start the conversation from this angle because they could provide a concrete example of the disease. A patient shared that he worried his friends might think he was lying or making up the disease because it seemed so strange. He explained: “*There are many who just don’t understand… and I often get the feeling that they think I’m lying or that I’m making it up, or that I don’t understand it myself or something… So that’s why I sometimes avoid it a little*”. (Patient 1).

Despite most families’ efforts to be open about the disease and its potential consequences, they often felt overwhelmed by the challenge of explaining ALD to friends and family. Families sometimes found themselves withdrawing from discussions about ALD due to the challenges it presented. Most describe this reluctance not as a desire to keep it a secret, but rather because it was too challenging to explain. Generally, parents in families where multiple family members had ALD experienced less loneliness because they had close family members going through similar experiences. Some parents also faced difficulties with authorities when seeking compensation for hospital visits, as officials struggled to understand why their seemingly healthy child required extensive follow-up: “*Well, they say they want to see him to assess how sick he is… They just can’t understand that he isn’t sick right now, and that is just insanely difficult to live with*” (Parent 3).

Patients especially described frustration when new doctors outside of the specialized units were unfamiliar with ALD. They explained how it made them feel even more isolated because even doctors were unfamiliar with the diagnosis: “*… For example, I was at a normal general practitioner and he really didn’t know what this was, because he was just a normal general practitioner. And I mean obviously, it feels lonely that even a doctor doesn’t know what it is*”. (Patient 2). This all contributed to a sense of disease-related loneliness.

## 5. Discussion

This study investigated the impact of the ALD diagnosis and the surveillance program on patients and their families prior to the onset of cALD. In the study, we observed a significant discrepancy between patients and their parents, with parents being more affected by and knowledgeable about the disease. However, despite this, both groups experienced disease-related loneliness.

From studying other chronic disorders it is known that having a child with a chronic disorder can lead to both physical and psychological health problems [27]. Although the parents in our study were thankful for the early diagnosis and had great trust in the healthcare professionals, it significantly impacted them, particularly in the time leading up to the follow-up consultation and while waiting for MRI results. This pattern has also been found amongst parents of children undergoing MRI surveillance for childhood brain cancer [22]. Here, it was also reported that the waiting period after the MRI scan until the results were given was very hard on the parents. Interestingly, a recent UK study [28] found that parents of children diagnosed with cALD almost felt a relief when the cALD diagnosis was eventually given because it meant something could now be done and the child could receive HCT, which underlines how stressful the waiting period can be for these families.

According to Lazarus’ transactional model of stress [29], a typical stress response involves seeking information about the stressor, which could explain the high level of knowledge observed in the parents. Parents also experience significant psychological stress when receiving the ALD diagnosis, which, as one parent suggested, could perhaps be alleviated by including follow-up sessions with a psychologist as part of the routine care. It would be important that such a follow-up was made by a psychologist with knowledge and experience in dealing with rare diseases.

Especially, the onset of cALD and the potential loss of their child were the major worries among the parental group. None of the parents had children older than 12 years, meaning that they were all still within the age range known to have a peak risk of cALD. We did not find that ALD patients themselves worried significantly about their diagnosis. One could speculate that the older patient group worried less about their disease because they were no longer within this age window. However, parental concerns extended beyond the diagnosis of cALD; they also harbored worries about their children’s future with AMN in adulthood, a trend that was not observed in the patient group to the same extent. Another factor may be the parents’ desire to shield their children from the anxieties associated with the diagnosis, a common theme observed among parents of children with various chronic diseases [30]. The patients that were interviewed in this study had all attended regular follow-ups since childhood. Due to the significant uncertainty and potential for life-threatening disease associated with the ALD diagnosis, it is natural that both parents and healthcare professionals want to protect the children from the burden of this knowledge. This dilemma of how and when to share information with young adult patients is found across other chronic diseases. In a Danish study from 2019 [30], it was also found that parents wanted to protect their chronically ill children from information they feared they might not be able to handle. However, as healthcare professionals, this is an area that can be improved. As our patients enter adolescence and adulthood, a special focus should be on how to inform these patients about their diagnosis in age-appropriate steps. This should be done in close cooperation with the parents and include an ongoing dialogue about what the parents feel is an appropriate level of information for their child. Especially, information about genetic counseling regarding future pregnancies was needed among the group of young adult patients with ALD diagnosis.

Patients with ALD generally maintained a positive outlook on their diagnosis. This is in line with other studies of patients under surveillance for potentially very serious diseases [20,23]. However, when occupied with their disease, it was generally very concrete issues that worried them. During childhood, the patients’ primary concern was discomfort from medical procedures such as blood draws, lying still in the MRI scan, or IV placements. The fear of needles is a common concern among children [31] and it becomes particularly challenging in managing chronic conditions, e.g., diabetes, which necessitate frequent venipunctures [32]. This issue has received considerable attention in recent years, leading to the development of various coping techniques [33,34,35]. There is a growing focus on incorporating play into hospital visits as a distraction for painful procedures, which is becoming more common in pediatric departments [36]. However, it is crucial to acknowledge that for children undergoing frequent ALD surveillance, fear of needles is a recurring fear that demands ongoing attention and intervention to ensure positive hospital experiences. Another concrete worry patients expressed was related to dietary restrictions in social settings. While no longer recommended as standard treatment in Denmark, those who had previously followed such restrictions or still did described challenges, especially in childhood, including feelings of isolation in social settings. Although it is a very different disease, a similar pattern has been seen in adolescents with familial hypercholesterolemia who also adhere to a diet specific to their diagnosis [37], which underlines that dietary restrictions in childhood and adolescence can have social implications that may require support to navigate.

In relation to this research, it is interesting to discuss the work by Timmermans et al. [38]. Timmermans et al. discuss “patients-in-waiting” who are detected by NBS but have an ambiguous or later onset diagnosis; thus, they are not actually sick at the time of diagnosis. They describe how patients-in-waiting exist in a state between healthy and sick, which is imposed by screening and surveillance aimed at secondary prevention. Their lives are usually characterized by lengthy processes and follow-up to address the diagnostic uncertainty. Timmermans et al.’s work explores how this affects patient identity and other aspects of their lives. What we observed in our study was not that the presymptomatic diagnosis had a major influence on the actual patients, but more so on the parents. The ALD patients diagnosed during childhood were successfully protected and supported by their parents who helped guide them through their “patient-in-waiting” status. However, many of the aspects described by Timmermans et al. were true for the parent group. This research suggests a nuance of the term “patients-in-waiting” directed at the parents, who become a type of patients-in-waiting-by-proxy.

Although this study found that parents were significantly more affected by their children’s ALD diagnosis and regular follow-ups than the children themselves, both parents and patients shared a common experience: the sense of disease-related loneliness accompanying the ALD diagnosis. Major contributors to this issue were the rarity and complexity of the disease. Both patients and parents expressed a desire to be transparent about the disease yet found it challenging to communicate the disease’s progression and potential consequences. This theme is consistent with other studies that emphasize the sense of loneliness associated with having a child with a rare disease [39,40]. From a healthcare perspective, one solution could involve providing parents and patients with clear and concise descriptions of the ALD diagnosis and surveillance program to enable their communication with others.

The focus of the study presented here was the long-term experience of the presymptomatic diagnosis and follow-up of patients with ALD, which is possible through either cascade testing or NBS. While several countries have implemented ALD in their NBS programs, few have explored the qualitative experiences of families receiving this diagnosis. Our study helps fill this gap by highlighting the challenges patients and parents face regarding early diagnosis of ALD. These insights can inform future decisions on including ALD in NBS programs, ensuring families receive early support and guidance to reduce stress.

One study from California [18] explored this experience by interviewing parents 4–26 months after their child’s diagnosis. The study revealed coping mechanisms and stressors similar to those found in our study. It showed that parents often used information-seeking to cope, while the uncertainty surrounding the ALD diagnosis remained a major source of stress. Another study [41] suggested that receiving the diagnosis through NBS could potentially reduce parental depression, indicating that early detection may help ease the psychological burden. Additionally, several studies [18,42] have shown that one of the most frustrating aspects for parents receiving a positive result for a metabolic disorder through NBS is the lack of adequate information during the initial contact. As we found in our study, even parents who have known about the ALD diagnosis for many years continue to struggle with understanding and explaining it. Therefore, when informing parents of a positive ALD screening, it is crucial that this is performed by a professional with a thorough understanding of the disease and enough time to explain it properly.

This qualitative study highlights several important aspects of how the asymptomatic ALD diagnosis affects patients and their families. However, there are some limitations to our study. Due to the rarity of the disease, the sample size was relatively small. Nonetheless, the responses to our interview questions were very consistent, which meant that we experienced patterns that suggest data saturation in both the patient and parent groups. Furthermore, the ALD patient group we interviewed was aged 18–25 and had all been in the ALD follow-up program since childhood. However, their thoughts about being in the program as children were subject to recall bias. Therefore, whenever possible, we tried to support these statements with input from parents who currently have children undergoing follow-up for ALD.

In the future, larger studies that also include the pediatric patient group and patients diagnosed through NBS would add valuable information to this area of research. Despite the small group of participants, this patient-centered research reveals nuances and patterns that warrant further exploration through more generalizable methods.

## 6. Conclusions

From this study, we have learned that while an early diagnosis of ALD and inclusion in the surveillance program is welcomed by parents and patients, this also imposes a significant psychological toll on the parents, who generally want to burden their children as little as possible with the disease. The patients, on the other hand, live relatively carefree lives despite their ALD diagnosis. A general theme of disease-related loneliness was present in both parents and patients. Overall, this work highlights that NBS for ALD in Denmark would have a greater psychological impact on parents, while patients may feel less burdened. Future focus areas to improve the experience of patients and parents in the ALD surveillance program and life with the diagnosis, in general, could include:

- Supporting patient transition from childhood to adulthood. Providing ALD patients in childhood and adolescence with comprehensive and age-appropriate information about their diagnosis for their adult lives, including genetic counseling for pregnancies. Encouraging parents to discuss ALD at home and providing them with strategies and tools to manage these discussions and questions.
- Incorporating routine psychological support for parents into the ALD surveillance program, provided by a psychologist with expertise in rare diseases.
- Prioritizing patient experience in childhood, with special attention to making painful procedures more manageable. This could include incorporating both pharmacological and non-pharmacological interventions.
- Providing tangible material about the ALD diagnosis to aid communication with friends and family. Help parents and patients to identify ALD patient organizations that can be used for support.
- Expediting MRI results and scheduling visits away from major holidays or significant life events.

## Figures and Tables

**Table 1:** Example of a code tree for the theme “Having a child with adrenoleukodystrophy (ALD) was a big psychological burden for parents”.

Main Theme	Subthemes	Initial Codes	Informants Quotes
Having a child with ALD was a big psychological burden for parents.	The worries were worst in the time around hospital visits and MRI. Parents coped by being well-informed about ALD. Parents avoided telling their children the difficult parts and carried this burden alone.	Worries are worst around the time of follow-up appointments. Fear of the result of the MRI scan. Fear of waiting for the phone call with follow-up results. Parents have sought information about ALD. Parents are well-informed about ALD. Parents worry about the disease. Parents avoid telling their children about the serious sides of ALD. Parents do not want to burden their children with the knowledge of ALD.	It’s difficult for me with his disease… And it has also caused me some periods of stress […] It takes up a lot of my thoughts… (Parent 7) It hits as soon as we start getting reminders in ”Min Sundhed” [The platform used for communicating with the healthcare system in Denmark], that’s when you get reminded… I find that I’m actually pretty good at not thinking about it in between [visits]… that’s about 4 months and then you’re reminded again. (Parent 3) It’s even worse when you’re waiting for results, and then when you get the results and they’re positive, you completely relax again. But then you also know that there will be another period where you’ll have to wait for new results again. (Parent 4) [Talking about when it is hardest] Definitely when we get closer to the periods where we have the follow-up appointment… Then we have to start preparing him and the siblings and ourselves. (Parent 7) [Talking about when it affects them the most] It’s usually around fall because that is when we have our follow-up appointments. So around that time, I start thinking about it more. (Parent 2) We’ve always planned it so that they called me, so I’ve always been the bearer of the news, both good and bad. So it’s me who waits around for the call. The first years I would stay home from work on those days, so that I wasn’t caught in a meeting or in a setting where I couldn’t receive bad news. (Parent 1) Well, I know that it comes in three forms. If we look at it for boys, during childhood that’s where the risk is biggest for these changes in the brain, which is very serious and that requires treatment, otherwise they won’t survive. Then there is the adrenal insufficiency which can hit, to my understanding, from when they are around two years old but also throughout adulthood, we can’t really say when that will hit. Then there is the more adult form which can affect a lot of different things such as the balance, difficulties with the legs… there are a lot of different things in the adult form, and that is also the form that women can be affected by. (Parent 2) We have told him that there might be problems with his legs and his adrenal glands, but we haven’t mentioned the brain specifically. My fear is that he starts googling ALD, because there are really some nasty details, and I would like to shield him from that […]. I would like to keep his hopes up”. (parent 5) We talk a lot about what is going to happen [talking about the procedures in the surveillance program], but not really why he has to or what comes from it … And we haven’t really talked about what the consequences are of getting a bad result from the scan. He doesn’t ask either. At some point, he probably will, and at that time we will have to make up our minds about how much we want to refer to his * [an affected relative’s] disease course. But otherwise, we’re taking it one step at a time and we try not to put words into his mouth or thoughts in his head that aren’t necessary. (Parent 2) It is also important to us that he’s allowed to be a completely normal child who doesn’t have to worry about anything. So we decided that when he asks about something, we’ll answer and try to give him the knowledge he needs. But otherwise, he doesn’t need to think about it—we’ll do that for him. (Parent 4)

**Table 2:** Demographics for participants. * Some parents had more than one child in the surveillance program. Parents declined participation for three sons aged 8–11; one son was not yet informed about the adrenoleukodystrophy (ALD) diagnosis; and for the other two, the parents believed the interview could be stressful for their children. HCT: hematopoietic cell transplantation.

Group	Number of Interviewed Participants	Age Range of ALD Patients	Patients Age at Diagnosis	Diagnostic Method	Patients’ Symptoms and Treatment
ALD patients over the age of 18	4	18–25 years	1–12	3 were diagnosed via cascade testing 1 was diagnosed due to onset Addison’s	1 patient had Addison’s disease and was treated with daily hormone substitution, none of the patients had symptoms of AMN, 3 were treated with Lorenzo’s oil throughout childhood, 2 of them were still taking Lorenzo’s oil.
ALD patients under the age of 18	0	Under 18	-	-	
Parents of ALD patients	7 parents *	3–11 years	0–3 years	Cascade testing	2 patients had adrenal insufficiency, 1 took daily hormone substitution, and 1 only when sick. 1 patient had recently undergone HCT at the time his parent was interviewed

**Table 3:** Overview of themes and subthemes identified. ALD; adrenoleukodystrophy.

Theme	Subtheme
ALD patients maintained mental resilience despite diagnosis and surveillance	Patients aspired to lead as normal lives as possible Patients had great trust in healthcare professionals and it gave them an opportunity to leave their own worries behind
Patients’ concerns matured with age and centered around situations that confronted them with their patient status	In childhood, patients primarily expressed concerns about concrete situations that were unavoidable due to their disease. The transition from child to adult was difficult Adult patients’ predominant concern arose when faced with the prospect of passing ALD to their children
Having a child with ALD was a big psychological burden for parents	Parent’s worries were worst in the time around hospital visits and MRI Parents coped by being well-informed about ALD Parents avoided telling their children the difficult parts and carried this burden alone
Parents of children with ALD had both short-term and long-term worries for their children’s health	
Due to its rarity, the diagnosis of ALD evoked a sense of isolation and disease-related loneliness	

## Data Availability

Data from this research are not available because they contain information that could compromise the privacy of research participants.

## Supplementary Materials

The following supporting information can be downloaded at: https://www.mdpi.com/article/10.3390/ijns10040073/s1, Interview guide for patients and parents: Table S1: Interview guide used for interviewing patients over the age of 18; and Table S2: Interview guide used for interviewing parents.
