# Supplementary material for: Psychological Impact of Presymptomatic X-Linked ALD Diagnosis and Surveillance: A Small Qualitative Study of Patient and Parent Experiences
Source: Int J Neonatal Screen. 2024 Oct 24;10(4):73. doi: 10.3390/ijns10040073 (PMC11587114; doi:10.3390/ijns10040073)
Supplement: Supplementary file 1 [file IJNS-10-00073-s001.zip › IJNS-3126233-supplementary.pdf]

Supplementary material

SECTION	QUESTIONS
WELCOME/ INTRODUCTION	Remember to provide water, tissues, and put a sign on the door to avoid interruptions.<br><br>Inform the participant that the interview will take between 45 minutes to an hour.<br><br>The purpose of the interview is to gain insight into your experience of being followed up here at the hospital and how it affects your life – this is something we know very little about today.<br><br>Written consent is checked. Inform the participant that the interview will be audio-recorded but will be transcribed immediately after the interview, and then the audio recording will be deleted. Both will be stored on a secure drive during the process, which only I have access to. All locations and names, etc., that can be traced back to the patient/you will be removed, so the patient/you cannot be recognized. The study will result in an article, where I might use quotes from our interview. These quotes will also be edited so that all identifying information (names, places, etc.) is removed or changed. Do you have any questions before we start the interview?
PATIENT'S UNDERSTANDING OF XALD AND THE FOLLOW-UP PROGRAM	Fist, I will ask you some questions about the disease and your visits here<br><br>What led you to start this follow-up program?<br><br>In your own words, can you explain why you are at the hospital today?<br><br>What can you tell me about the disease ALD?<br><br>What are your thoughts on the consequences of the tests being done today?<br><br>What do you know about the treatment options for XALD?
PATIENT'S THOUGHTS ON THE FOLLOW-UP PROGRAM	Now, here are some questions specifically about how you experience being followed up here:<br><br>What are your thoughts on the tests you have to undergo today (and their purpose)?<br><br>Have you felt prepared for the tests you need to have done at the hospital?<br><br>What thoughts have you had about the tests beforehand?

	Have your thoughts changed over the years you have been undergoing the tests? If yes, can you describe how? What do you think might be the reason for this? (You could explore factors like age, the illness of a family member, etc.)<br><br>Are there periods during the year when you think more about the tests than at other times? If yes, what can you tell me about that? Why do you think this is?<br><br>*Others with ALD in this follow-up program have described it as feeling lonely. What do you think about that?*
CLARIFYING HOW XALD AFFECTS THE PATIENT'S LIFE	The next questions are about your journey here over several years from childhood to adulthood:<br><br>During the periods between hospital visits, how are you affected by having this diagnosis?<br><br>What thoughts do you have about having a disease? Can you tell me more about them?<br><br>*ALD has been described by other patients as an invisible disease. What do you think about that? (Optionally supplemented with "How does it affect you that it is an invisible disease?" if they do not bring it up themselves.)*<br><br>What is your earliest memory of the tests and the disease? The first thing you can remember? (And ask follow-up questions based on the response.)<br><br>How were you introduced to the diagnosis as a child, do you remember it?<br><br>What thoughts did you have about the tests and the disease when you were a child?<br><br>How has your experience of the diagnosis XALD and the follow-up program, i.e., the tests here, changed as you have grown older?<br><br>Have you felt that your diagnosis with XALD makes you different from others? How? Does it have any consequences for you? (Ask follow-up questions and be mindful of, e.g., tendencies towards isolation or symptoms of psychopathology.)<br><br>How do you talk to friends and family about the tests/disease? What do you talk about?<br><br>- If the patient mentions symptoms of depression or anxiety, these can be explored by openly asking about the specific symptom (when it occurs, if it is still present, if it is always there or only in specific situations/times. Additionally, questions about the remaining symptoms of

	anxiety/depression will be asked. There is a protocol for how to act if it is suspected that a parent or patient shows signs of severe mental distress-
CONCLUSION OF THE INTERVIEW	Do you feel that you have received the support you needed in the diagnosis and follow-up process? If not, what have you needed that was not provided?<br><br>Is there anything you would like to see done differently in your process here?<br><br>The interview is almost finished, and I have no more questions. Is there anything you would like to add?

Supplementary Table S1: interview guide used for interviewing patients over the age of 18

SECTION	**QUESTIONS**
WELCOME/ INTRODUCTION	Remember to provide water, napkins, and put a sign on the door to avoid disturbances.<br><br>Inform the participant that the interview will take between 45 minutes to an hour. The purpose of the interview is to gain insight into your experience of being followed up here at the hospital and how it affects your life – something we know very little about today.<br><br>Written consent is checked. Inform the participant that the interview will be audio-recorded but will be transcribed immediately after the interview, and then the audio recording will be deleted. Both will be stored on a secure drive during the process, which only I have access to. All locations and names, etc., that can be traced back to the patient/you will be removed, so the patient/you cannot be recognized. The study will result in an article, where I might use quotes from our interview. These quotes will also be edited so that all identifying information (names, places, etc.) is removed or changed. Do you have any questions before we start the interview?
PARENTS' UNDERSTANDING OF XALD AND THE FOLLOW-UP PROGRAM	First, I will ask you some questions about the disease and your visits here:<br><br>In your own words, could you tell me what led to your child starting this follow-up program?<br><br>Could you explain your understanding of the purpose of the tests your child will undergo today?<br><br>What can you tell me about the disease ALD?

	What do you know about the treatment options available for XALD?
PARENTS' THOUGHTS ON THE FOLLOW-UP PROGRAM AND ALD	Now, some questions specifically about how you experience the follow-up program:<br><br>Leading up to today, what thoughts have you had about the tests your child will undergo?<br><br>Have you felt prepared for the tests your child will undergo today?<br><br>How have you talked to your child about the tests that were to be performed?<br><br>How have you talked to your child about ALD?<br><br>Are there periods during the year when you think more about the hospital visits and tests than other periods? If so, why do you think that is?<br><br>In the periods between hospital visits, how does your family cope with your child having this diagnosis?<br><br>Does your child have siblings? If yes: What challenges does this present in relation to the diagnosis?<br><br>*Others with children with ALD in this follow-up program have described that it can feel lonely, what are your thoughts about that?*
CLARIFICATION OF HOW XALD AFFECTS THE PATIENT'S LIFE	What thoughts do you have about your child having a diagnosis?<br><br>Does your child face challenges that other children do not? If yes, what? How do you handle them? How do you feel your child is overall compared to other children?<br><br>How do you talk to your family/friends about the disease/follow-up here and the tests? Follow-up: what do you talk about? Have you considered how you should handle the conversation/approach it?<br><br>How do the hospital visits affect your child?<br><br>What thoughts does your child have about having a diagnosis?<br><br>How does your child talk to family/friends about the disease/tests at the hospital?<br><br>What thoughts do you have about your own and your child's future with the diagnosis?

	Other parents have described ALD as an invisible disease, what do you think about that? (Possibly supplemented with "How does it affect you that it is an invisible disease?" if they don't mention it themselves)<br><br>-If parents mention symptoms of depression or anxiety in themselves or the child, this can be explored by openly asking about the specifics. There is a protocol for how to act if it is suspected that a parent or child shows signs of severe mental distress-
CONCLUSION OF THE INTERVIEW	Do you feel you have received the support you needed in the diagnosis and follow-up process? If not, what have you needed that has not been met?<br><br>Is there anything you would like to see/change in the process here?<br><br>The interview is almost over, and I have no further questions. Is there anything you would like to add?

Supplementary Table S2: interview guide used for interviewing parents
